# The Correlation Analysis of Two Common Polymorphisms in *STAT6* Gene and the Risk of Asthma: A Meta-Analysis

**DOI:** 10.1371/journal.pone.0067657

**Published:** 2013-07-04

**Authors:** Li Zhu, Qingqing Zhu, Xinlin Zhang, Hongwei Wang

**Affiliations:** 1 Center for Translational Medicine and Jiangsu Key Laboratory of Molecular Medicine, Medical School of Nanjing University, Nanjing, Jiangsu Province, China; 2 Department of Cardiology, Drum Tower Hospital, Medical School of Nanjing University, Nanjing, Jiangsu Province, China; University of Iowa, United States of America

## Abstract

**Background:**

Several studies have reported that the *GT* dinucleotide repeat length polymorphism and the *G2964A* polymorphism in *signal transducer and activator of transcriptional factor 6* gene are associated with asthma susceptibility, but others have conflicting results. Our meta-analysis aimed to elucidate the emerging paradigms.

**Methods:**

We searched PUBMED, EMBASE, ISI web of knowledge, Chinese National Knowledge Infrastructure, and Wanfang databases. Odds ratios (ORs) and 95% confidence intervals (95% CIs) were used to evaluate the strength of association. We applied Bonferroni step-down and Benjamini-Hochberg step-up methods to adjust the values for multiple comparisons.

**Results:**

A total of 12 individual studies in 11 articles were included in the meta-analysis. For *GT* repeat polymorphism, the *S* allele had approximately 45% increased risk of asthma (*S* vs. *L*: OR = 1.45, 95% CI = 1.22–1.71, *P*_UNCORRECTED_ <0.001, *P_Bon_* <0.001, *P_FDR_* <0.001). Further analysis indicated that *GT_13_* and *GT_14_* contributed to asthma risk, whereas *GT_15_* and *GT_16_* were protective (*GT_13_* vs. *GT_15_*: OR = 1.38, 95% CI = 1.16–1.65, *P*_UNCORRECTED = _0.001, *P_Bon = _*0.005, *P_FDR = _*0.002). Similar results were obtained in the subgroup analysis of Asian population. *G2964A* polymorphism analysis showed that the *AA* genotype moderately increased the risk of asthma by 47% compared with the *GG* genotype (OR = 1.47, p = 0.068) in Chinese population, whereas the *2964A* allele moderately increased the risk of asthma in Chinese population by 18% (*2964A* vs. *2964G*: OR = 1.18, p = 0.08). However, none of the associations reached statistically significant levels particularly after correction for multiple testing.

**Conclusions:**

This meta-analysis suggests that *S* allele (*GT_13_* and *GT_14_*) of the *GT* repeat polymorphism confers significant risks to asthma. However, the *G2964A* polymorphism does not have an association with the susceptibility to asthma.

## Introduction

Asthma is a complex and chronic respiratory disorder characterized by hyper responsiveness, obstruction, and chronic inflammation of the airway. Several studies have strongly indicated that asthma has a hereditary origin considering that asthma and asthma-related traits have been observed in families [1]. Studies have also investigated the genetic association of asthma by using “genome-wide linkage” and “candidate-gene association” methods. More than 10 gene regions have been associated with asthma or asthma-related phenotypes [2], [3]. Most of these identified candidate genes encode molecules involved in innate immunity, T helper 2 (Th2) cell differentiation, lung function regulation, and airway remodeling; other genes are associated with epithelial biology and mucosal immunity [4]. Among these candidate genes, *signal transducer and activator of transcription factor 6* (*STAT6*) is one of the most widely investigated important genes [5]–[15].

*STAT6* gene is located at 12q13.314.1 [16], one of the most susceptible regions associated with asthma. *STAT6* is normally activated by Th2-related cytokines such as interleukin-4 (IL-4) and IL-13 [17]. *STAT6* is activated to induce Th2 cell differentiation from naïve T cells and IgE production [18]. Activated *STAT6* also regulates the expression of Th2 chemokines [19] that function as asthma indicators when these chemokines are upregulated [20]. Studies have reported the association between *STAT6* gene polymorphisms and the susceptibility to asthma in various populations. Polymorphisms or haplotypes associated with asthma or serum IgE levels have also been reported [5], [21], [22]. Among these polymorphisms, *GT* dinucleotide repeat length polymorphism in exon 1 of *STAT6* and *G2964A* polymorphism in the 3′-untranslated region are two of the most commonly investigated, but no inclusive result has been obtained in both polymorphisms. Different genotyping methods, a relatively small sample size, and population differences may partly account for such discrepancies.

In this study, a meta-analysis was performed to delineate the association of these two polymorphisms (*GT* repeat length polymorphism and *G2964A* polymorphism) with the risk of asthma based on case-control studies. To the best of our knowledge, this study is the first genetic meta-analysis to identify the association between the *STAT6* gene polymorphism and the risk of asthma.

## Materials and Methods

### Search Strategy

Two investigators (LZ and QZ) independently performed the abstract screening, full text review, and data collection by using a computer-based approach. Studies published online were searched in PUBMED, EMBASE, ISI web of knowledge, Chinese National Knowledge Infrastructure (CNKI), and Wanfang databases with the following search terms: “*STAT6*” or “signal transducer and activator of transcription”, “asthma” or “asthmatic”, and “polymorphism,” “mutationm” or “variation”. The publication date was restricted to the latest search on December 8, 2012. No language restriction was imposed. Reference lists of the retrieved records were manually screened and reviewed for other relevant publications.

### Study Selection

The records included in the meta-analysis were case-control studies. Family or sibling pair-based designs were excluded. All of the studies investigated the association between *STAT6* polymorphisms (*GT* dinucleotide repeat length polymorphism and/or *G2964A*) and the risk of asthma. Allelic or genotypic distributions of both cases and controls were available. All types of asthma, including atopic or non-atopic were included regardless of the age of the investigated population. Some studies also included mixed types of allergic diseases such as atopic dermatitis; however, these studies were excluded because the number of asthma patients for each genotype or allele is unavailable. Among the studies with overlapping investigated population, only the study with the most extensive data was considered. Animal studies were excluded.

### Data Extraction

All of the relevant records were initially scanned based on titles and abstracts. Studies that did not satisfy the aforementioned criteria were rejected. The remaining studies were evaluated after a full text review was conducted. Two authors (LZ and QZ) independently extracted data from the included records. A final agreement was achieved after a consensus meeting (LZ, QZ, XZ, and HW *et al.*). The following data were obtained from each eligible study: first author’s name, year of publication, country of origin, ethnicity, age group of the study population, definitions of asthma and control, number of participants in the case group and the control group, and research methods for genotyping.

### Statistical Analysis

Hardy-Weinberg distribution of genotypes of the polymorphisms in *STAT6* gene was assessed in the control group using the χ^2^ test, p<0.05 was considered significantly different. Odds ratios (ORs) and 95% confidence intervals (CIs) were used to evaluate the strength of association between the *STAT6* polymorphisms and the risk of asthma. In our study, only dichotomous data analysis was performed, and the data were presented as numbers of different genotypes or alleles in different groups. For the *GT* dinucleotide repeat length polymorphism, the alleles with repeat numbers 12, 13, 14, 15, 16, and 17 were simplified as *GT_12_*, *GT_13_*, *GT_14_*, *GT_15_*, *GT_16_*, and *GT_17_*, respectively. A cut-off point was set between *GT_14_* and *GT_15_*. Alleles ≤14 were defined as short alleles (*S*) and alleles ≥15 were regarded as long alleles (*L*). Pooled ORs and their corresponding 95% CIs were generated to compare allelic frequencies (*GT* repeat length polymorphism: *S* vs. *L*, *GT_13_* vs. *GT_14_*, *GT_13_* vs. *GT_15_*, *GT_15_* vs. *GT_16_*; *G2964A*: *2964A* vs. *2964G* in the Chinese population) and genotype frequencies with multiple genetic models (*G2964A*, recessive model: *GG* vs. *AA*+*AG*; dominant model: *AG*+*GG* vs. *AA*; additive model: *AA* vs. *AG*, *AA* vs. *GG*, *AG* vs. *GG*). The significance of pooled ORs was determined with a Z test. Heterogeneity was evaluated with the χ^2^-based Q test and I^2^ index [23], p<0.10 suggests significant heterogeneity. A random-effect model (Dersimonian and Laird method) was used [24]; otherwise, a fixed-effect model (Mantel-Haenszel method) was used [25]. Sensitivity analysis was performed by omitting one study at each time to evaluate the influence of an individual study on the overall effect. Studies that deviated from Hardy-Weinberg equilibrium were also included in the sensitivity analysis. Begg’s and Egger’s tests were performed to assess the publication bias, p<0.05 indicates a significant publication bias [26], [27]. To adjust the values for multiple comparisons, we used Bonferroni step-down (Holm) correction and Benjamini-Hochberg (BH) step-up correction methods, which control the family-wise error rate and false discovery rate (FDR), respectively. Statistical analysis was performed using STATA version 11.0 software (STATA Corporation, College Station, TX, USA).

## Results

### Study Characteristics

**Figure 1:**
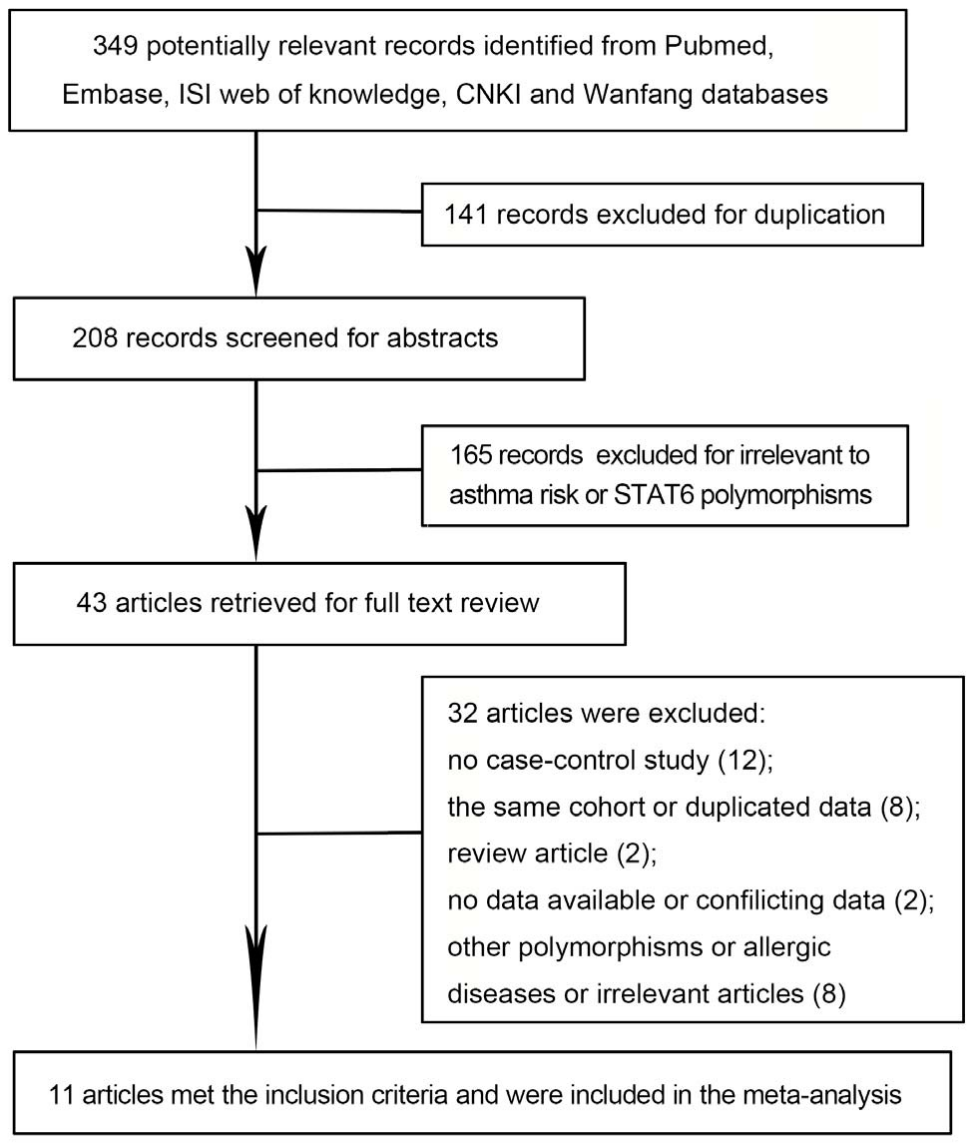
Flow diagram of the inclusion and exclusion of published articles in the meta-analysis.

A total of 349 publications were initially identified from PUBMED, EMBASE, ISI web of knowledge, CNKI, and Wanfang databases. After titles and abstracts were screened, 306 articles were excluded because of duplication or irrelevance to the meta-analysis. The full texts of the remaining 43 records were carefully reviewed and 31 of these records were discarded because of several factors (**Fig. 1**). Among these articles, one conference report was excluded because of unavailable genotype data [28]. Another article was also excluded because of inconsistent data [29] regarding *STAT6 G2964A* polymorphism. One article investigated two cohorts of population, and each cohort is regarded as two separate case-control studies [5]. Twelve case-control studies presented in eleven articles satisfied the inclusion criteria and thus were included in the meta-analysis [5]–[15]. Among these case-control studies, five studies described *GT* dinucleotide repeat length polymorphism [11]–[15] and seven studies investigated *G2964A* polymorphism [5]–[10]. Among the studies on *GT* repeat length polymorphism, four studies involved Asian populations, in which two focused on Chinese populations [14], [15] and the remaining two considered Japanese populations [12], [13]. One study focused on a Caucasian population specifically the British population [11]. Among the studies on *G2964A* polymorphism, four studies were performed on a Chinese population [7]–[10], two studies focused on a Japanese cohort [6], [11], and one study described a Caucasian population [11]. Only the studies on Chinese populations provided a detailed number of each genotype (*AA*, *AG*, and *GG*) in case and control groups. For the studies on Japanese and Caucasian cohorts, a recessive model was considered and only data of *AA*+*AG* and *GG* genotypes were available. Age was not considered as a restricted variable. Children/adults or both populations were included in the studies. However, three studies did not provide a detailed age group of the population investigated [5], [11], [13]. Polymerase chain reaction-based genotyping methods were provided in all of the studies (**Table 1**).

**Table 1:** Descriptions of studies included in the meta-analysis.

First author	Year	Country	Ethnicity	Age group	Asthma (n)	Control (n)	Genotyping method
Gao *et al.*	2000	UK	Caucasian	NA	181	150	PCR-sequencing
Gao *et al.*	2000	UK	Asian	Children/adults	400	100	PCR-sequencing
Tamura *et al.*	2002	Japan	Asian	Children	73	66	PCR-SSCP
Hu *et al.*	2005	Chinese	Asian	Children/adults	120	112	PCR-RFLP
Li *et al.*	2007	Chinese	Asian	Adults	95	95	PCR-SSCP
Ding *et al.*	2010	Chinese	Asian	Adults	108	115	PCR-RFLP
Lin *et al.*	2011	Chinese	Asian	Children	113	87	PCR-RFLP
Gao *et al.*	2004	UK	Caucasian	NA	78	136	PCR-CE
Shao *et al.*	2004	Janpan	Asian	NA	114	172	PCR-CE
Suzuki *et al.*	2004	Japan	Asian	Children/adults	298	166	PCR-CE
Hu *et al.*	2005	Chinese	Asian	Children/adults	135	109	PCR-STR
Wang *et al.*	2011	Chinese	Asian	Children	107	96	PCR-sequencing

**Abbreviations:** NA: not available; PCR: Polymerase Chain Reaction; SSCP: Single Strand Conformation Polymorphism; RFLP: Restriction Fragment Length Polymorphism; CE: Capillary Electrophoresis; STR: Short Tandem Repeat.

### Meta-Analysis of Association between the *STAT6 GT* Repeat Length Polymorphism and the Risk of Asthma

**Figure 2:**
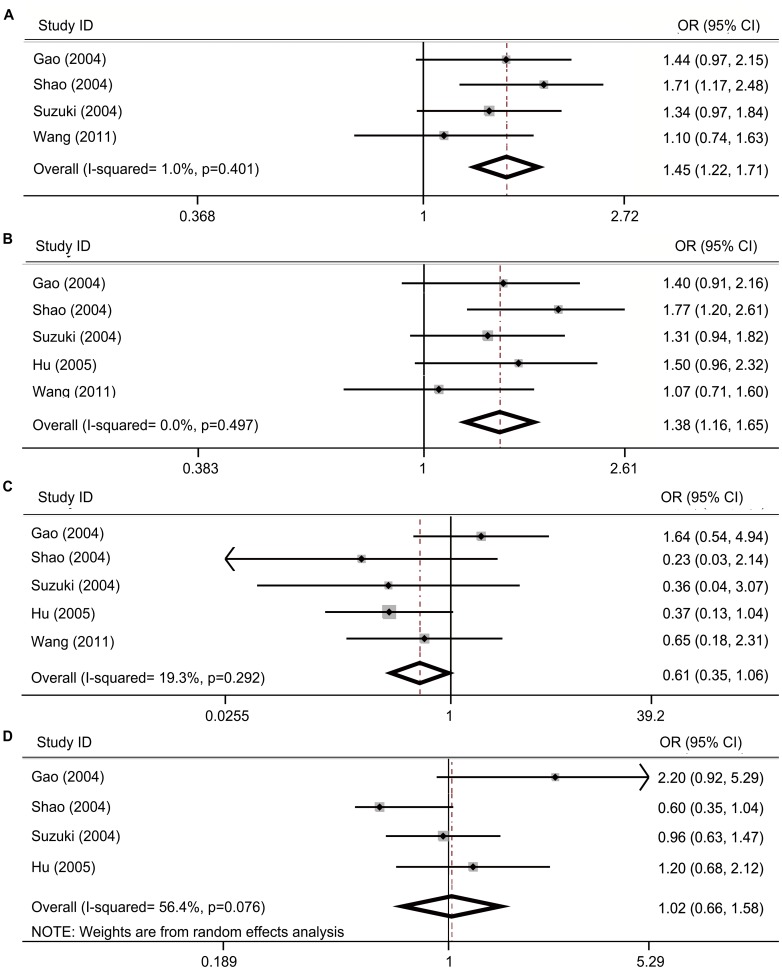
Association between *GT* repeat length polymorphism in *STAT6* with the risk of asthma. The results were shown by forest plots, each study is shown by the first author name, year of publication, individual and overall ORs (odds ratio) and 95% CI (confidence intervals). Box and horizontal line represent OR and 95% CI of the corresponding study, and the diamond represents the overall OR and 95% CI. Alleles ≤14 were defined as short alleles (*S*), and alleles ≥15 were regarded as long alleles (*L*). (A) *S* vs. *L* in all the populations, fixed-effects model; (B) *GT_13_* vs. *GT_15_* in all the populations, fixed-effects model; (C) *GT_13_* vs. *GT_14_* in all the populations, fixed-effects model; (D) *GT_15_* vs. *GT_16_* in all the populations, random-effects model.

A total of five studies describing the association between the *STAT6 GT* polymorphism and the risk of asthma were enrolled in the meta-analysis [11]–[15]. Among these studies, one study focused on a Caucasian population [11] and the remaining four studies described Asian populations [12]–[15]. For the *GT* repeat length polymorphism in *STAT6*, no consensus of the optimal cut-off point was achieved to classify the alleles. In this meta-analysis, the cut-off point was set between 14 and 15. The alleles were then divided in two groups: short allele (*S* ≤14) and long allele (*L* ≥15). *GT_13_* and *GT_15_* were the two most common alleles found and categorized in the *S* group and the *L* group, respectively, allowing balance to the number of each group. The comparison of *S* allele and *L* allele between cases and controls revealed a significant association of the *S* allele and the risk of asthma (OR = 1.45, 95% CI = 1.22–1.71, *P*_UNCORRECTED_ <0.001, *P_Bon_* <0.001, *P_FDR_* <0.001; **Table 2**, **Fig. 2A**). To identify the exact allele that contributed to the susceptibility of asthma, we performed the meta-analysis by conducting allele-allele comparisons, including *GT_13_* vs. *GT_15_*, *GT_13_* vs. *GT_14_*, and *GT_15_* vs. *GT_16_*. Our results indicated that a significant association was found in *GT_13_* vs. *GT_15_* (OR = 1.38, 95% CI = 1.16–1.65, *P*_UNCORRECTED = _0.001, *P_Bon = _*0.005, *P_FDR = _*0.002; **Table 2**, **Fig. 2B**). A moderate difference was found in *GT_13_* vs. *GT_14_* (OR = 0.61, 95% CI = 0.35–1.06; **Table 2**, **Fig. 2C**). No association was detected in *GT_15_* vs. *GT_16_* between the cases and controls (OR = 1.02, 95% CI = 0.66–1.58; **Table 2**, **Fig. 2D**). The meta-analysis results suggested that *GT_13_* and *GT_14_* were more prevalent in asthmatic patients compared with the control. These carriers could improve the risk of asthma, whereas *GT_15_* and *GT_16_* may provide protection against the development of asthma. Given that *GT_13_* and *GT_15_* were the two most common alleles in asthmatic patients and control subjects, we proposed that *GT_13_* was a risk allele of asthma and *GT_15_* allele was a protective allele. Subgroup analysis of the Asian population showed similar results (**Table 2**). Publication biases of all the meta-analysis were evaluated by Begg’s and Egger’s tests. No significant bias was detected (**Table S1**).

**Table 2:** Summary of meta-analysis results on *GT* dinucleotide repeat length polymorphism in *STAT6* gene.

	OR	95% CI	*p* value	Bon	FDR
**Overall**					
*S* vs. *L*	1.45	1.22–1.71	<0.001	<0.001	<0.001
*GT_13_* vs. *GT_15_*	1.38	1.16–1.65	<0.001	0.002	<0.001
*GT_13_* vs. *GT_14_*	0.61	0.35–1.06	0.080	0.240	0.107
*GT_15_* vs. *GT_16_*	1.02	0.66–1.58	0.912	0.912	0.912
**Asian population**					
*S vs. L*	1.45	1.20–1.74	<0.001	<0.001	<0.001
*GT13 vs. GT15*	1.38	1.14–1.67	0.001	0.005	0.002
*GT13 vs. GT14*	0.41	0.20–0.83	0.013	0.053	0.021
*GT15 vs. GT16*	0.90	0.67–1.19	0.450	0.899	0.514

OR: odds ratio; 95% CI: 95% confidence interval; Bon: *p* value in stepdown Bonferroni testing; FDR: *p* value from Benjamini- Hochberg method control for false discovery rate (FDR); *S*: short allele, allele ≤14 *GT* repeats; *L*: long allele, allele ≥15 *GT* repeats; *GT_13_*, *GT_14_*, *GT_15_*, *GT_16_* were abbreviations of the alleles with repeat number 13, 14, 15 and 16 respectively.

### Meta-Analysis of Association between the *STAT6 G2964A* Polymorphism and the Risk of Asthma

**Figure 3:**
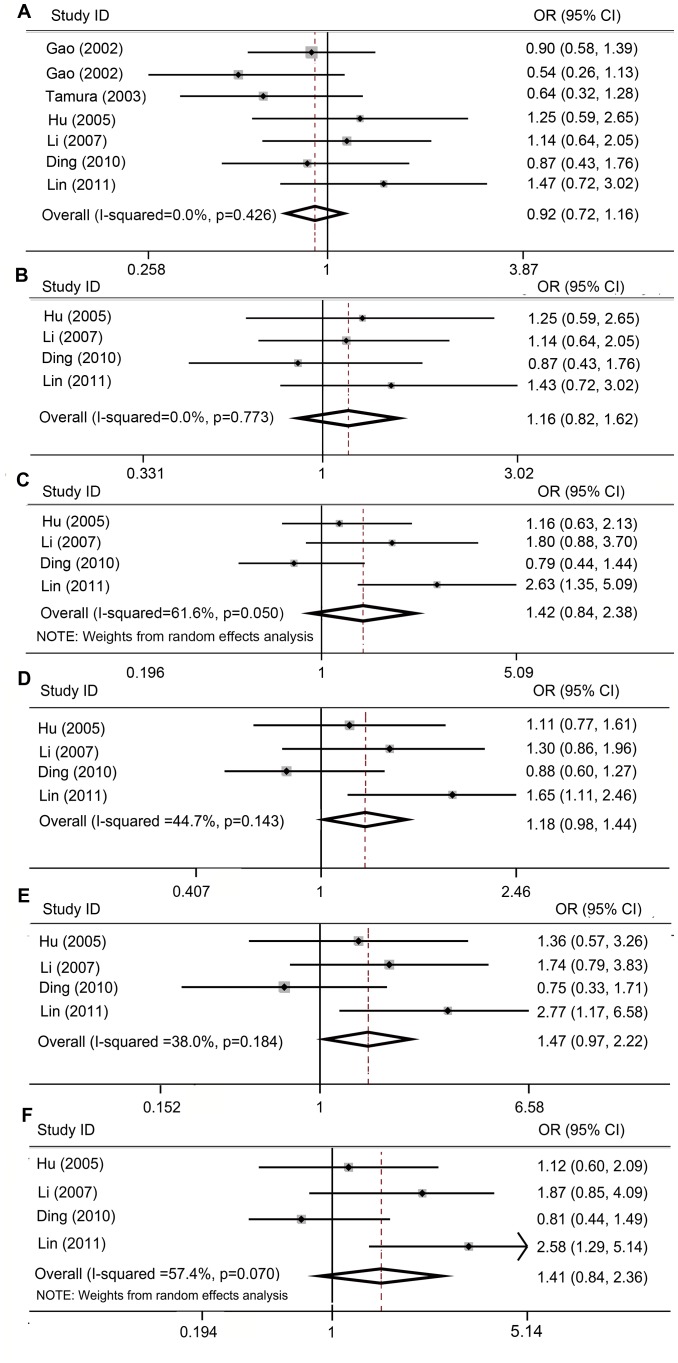
Association between *G2964A* polymorphism in *STAT6* with the risk of asthma. Each study is shown by the first author name, year of publication, individual and overall ORs (odds ratio) and 95% CI (confidence intervals). Box and horizontal line represent OR and 95% CI of the corresponding study, and the diamond represents the overall OR and 95% CI. (A) *AA*+*AG* vs. *GG* (recessive model) in all the populations, fixed-effects model; (B) *AA*+*AG* vs. *GG* in Chinese population, fixed-effects model; (C) *AA* vs. *AG*+*GG* (dominant model) in Chinese population, random-effects model; (D) *A* vs. *G* in Chinese populations, fixed-effects model; (E) *AA* vs. *GG* in Chinese population, fixed-effects model; (F) *AA* vs. *AG* in Chinese population, random-effects model.

Six articles presenting seven studies have demonstrated the association between the *STAT6 G2964A* polymorphism and susceptibility to asthma [5]–[10]. For the meta-analysis of the recessive genetic model (*AA*+*AG* vs. *GG*), no significant association was detected, with a pooled OR = 0.92 and 95% CI = 0.72–1.16 (**Table 3**; **Fig. 3A**). Low between-study heterogeneity (I^2^ = 0.0%, p = 0.426) and no significant publication bias (p = 1.00, 0.959 for Begg’s test and Egger’s test, respectively) were found. Sensitivity analysis yielded no significant change. Among the seven studies, four were performed in Chinese population and provided a detailed number of each genotype (*AA*, *AG*, and *GG*). A subgroup analysis of the Chinese population was also performed. Similarly, no significant result was obtained after comparison using the recessive models (*AA*+*AG* vs. *GG*), with OR = 1.16 and 95% CI = 0.82–1.62 (**Table 3**; **Fig. 3B**). After the dominant model (*AA* vs. *AG*+*GG*) was used, no significant association was observed with OR = 1.42 and 95% CI = 0.84–2.38 in a random-effect model (**Table 3**; **Fig. 3C**). Moderate associations were found between the allele *2964A*/genotype *AA* and the risk of asthma (2964*A* vs. 2964*G*: OR = 1.18, 95% CI = 0.98–1.44, *P*_UNCORRECTED = _0.084, *P_FDR = _*0.140; *AA* vs. *GG*: OR = 1.47, 95% CI = 0.97–2.22, *P*_UNCORRECTED = _0.068, *P_FDR = _*0.135; **Table 3**; **Figs. 3D and 3E**). However, both associations were not strong enough to reach statistically significant levels. No association was found between *AA* and *AG* genotypes (*AA* vs. *AG*: OR = 1.41, 95% CI = 0.84–2.36, random-effect model; **Table 3**; **Fig. 3F**). Results of analyses after the study of Ding *et al*. was excluded are shown in **Table 3** and **Fig. S1**. However, the study of Ding *et al*. satisfied the inclusion criteria and was in Hardy-Weinberg equilibrium. Thus, the results should be carefully interpreted after this particular study was excluded. Publication biases of meta-analysis were evaluated by Begg’s and Egger’s tests. Accordingly, no significant bias was detected (**Table S2**). All of the studies except the study of Hu [10] were in Hardy-Weinberg equilibrium. After this study was excluded, sensitivity analysis showed statistically similar results in all of the analyses (**Table S2**).

**Table 3:** Summary of meta-analysis results on *G2964A* polymorphism in *STAT6* gene.

	OR	95% CI	*p* value	Bon	FDR
**Overall**					
*AA*+*AG vs. GG*	0.92	0.72–1.16	0.464	0.798	0.464
**Chinese population**					
*AA*+*AG vs. GG*	1.16	0.82–1.62	0.399	0.798	0.443
*AA vs. AG*+*GG*	1.42	0.84–2.38	0.189	0.755	0.246
*AA vs. GG*	1.47	0.97–2.22	0.068	0.405	0.135
*AA vs. AG*	1.41	0.84–2.36	0.197	0.755	0.246
*A vs. G*	1.18	0.98–1.44	0.084	0.420	0.140
**Work of Ding removed**					
*AA vs. AG*+*GG*	1.73	1.19–2.52	0.004	0.044	0.038
*AA vs. GG*	1.86	1.15–3.02	0.012	0.094	0.038
*AA vs. AG*	1.69	1.14–2.51	0.009	0.079	0.038
*A vs. G*	1.32	1.06–1.66	0.015	0.105	0.038

OR: odds ratio; 95% CI: 95% confidence interval; Bon: *p* value in stepdown Bonferroni testing; FDR: *p* value from Benjamini- Hochberg method control for false discovery rate (FDR).

## Discussion

The present meta-analysis considered 12 case-control studies in 11 articles and was the first meta-analysis report to investigate the association between the two common polymorphisms in *STAT6* and the susceptibility to asthma. For the *GT* dinucleotide repeat length polymorphism in exon 1 of *STAT6*, the *S* allele (≤14 repeats) and the *L* allele (≥15 repeats) were compared, suggested a significantly strong association between the *S* allele and the susceptibility to asthma. The *S* allele had approximately 45% increased risk of asthma. Pair-wise comparisons showed that the *GT_13_* allele significantly increased the risk of asthma by 38% compared with the *GT_15_* allele. The *GT_14_* allele was identified as an allele with a higher risk, which moderately increased the risk of asthma compared with the *GT_13_* allele. No significant difference was observed between *GT_15_* and *GT_16_* alleles. Given that the frequencies of *GT_14_* and *GT_16_* alleles were extremely lower than those of *GT_13_* and *GT_15_* alleles, we considered *GT_13_* and *GT_15_* as the most dominant risk alleles of asthma for *STAT6 GT* repeat polymorphism.

Various studies have investigated the *STAT6 GT* repeat polymorphism and the risk of asthma. For instance, Gao *et al*. [11] reported that the frequency of *GT_13_* allele is significantly increased in atopic asthmatic subjects, but the frequency of *GT_16_* allele is inversely correlated with asthma, indicating that *GT_13_* allele may be a risk allele and *GT_16_* allele is likely a protective allele of asthma. Suzuki *et al*. [12] reported that the frequency of *GT_15_* allele is lower in asthmatic patients, but the frequencies of short alleles *GT_12_*, *GT_13_*, and *GT_14_* are higher. Shao *et al*. [13] also found a significant difference in allelic distributions between patients and control subjects, but the frequency of *GT_15_* allele is the only difference detected. Other studies have also reported negative results [14] or even opposite conclusions [30]. In our meta-analysis, a significant distribution difference was observed in *S* allele vs. *L* allele between asthma cases and controls. The mechanisms by which this polymorphism affects asthma could be possibly determined based on the previously published studies. *GT* dinucleotide repeat variant is located in exon 1, which is a coding region found in the 5′-untranslated region of *STAT6* gene [16]. Several studies have shown that the 5′-untranslated region can be involved in the regulation of *STAT6* gene expression. For instance, Gao *et al*. [11] found that patients with *GT_13_* allele have significantly higher levels of total serum IgE, an important marker of asthma, than those with *GT_16_* allele. Therefore, *GT_13_* allele may increase the risk of asthma by upregulating the production of total serum IgE [11] probably by a synergistic effect with *2964A* allele [6]. Gao *et al*. [11] further found that *GT_13_* allele can significantly increase the promoter activity of *STAT6* gene probably by binding to transcriptional silencers and by forming non-β-form DNA conformations. As a result, *STAT6* gene expression is increased and asthma is induced via an IL-4/IL-13 pathway.

For *G2964A* polymorphism, the overall effect observed in a recessive genetic model (*AA*+*AG* vs. *GG*) and a dominant model (*AA* vs. *AG*+*GG*) did not show significant correlation with asthma. After pair-wise comparisons were performed, *AA* homozygote carriers moderately increased the risk of asthma by 47% compared with the *GG* homozygote in Chinese population. Further analysis of the allele studies revealed that the *2964A* allele moderately increased the risk of asthma by 18% in the Chinese cohort. However, none of the comparisons generated statistically significant results.

Previous studies on the *G2964A* polymorphism and the risk of asthma were inconsistent and inconclusive. For instance, a strong association between mild atopic asthma and this polymorphism is observed in a Japanese population but not in a British population [5]. Lin *et al*. [8] found a significant association of this polymorphism in a Chinese cohort, suggesting that the *2964A* allele is a risk allele and the *AA* homozygote is a risk genotype to asthma. However, no association was found in replicated studies in either a Japanese population [6], [31] or a Caucasian population in a pair-sib study [30]. The difference in allergic phenotypes may be partly accounted for the discrepancy. The enrolled populations in the studies of Gao *et al*. [5] and Lin *et al*. [8] comprised asthmatic patients. Other allergic phenotypes alongside with asthma are also included in replicated studies [31]. The difference in ethnic populations may be another reason.

Genome-wide association (GWA) studies on asthma have suggested that several polymorphisms in several genes, including *ORMDL3-GSDMB*, *DENND1B*, *HLA-DP*, *SLC30A8*, *IL1RL1-IL18R1*, *IL33*, *SMAD3*, *TSLP*, and *NOTCH4* [32]–[35] are associated with asthma. However, none of these GWA studies revealed any association of the variants in *STAT6* gene with asthma. The *GT* repeat variant has not been assayed on GWAS microarrays in these studies. To the best of our knowledge, no study has attempted to determine whether or not any SNP is in strong disequilibrium with this variant. A good proxy SNP of the *GT* variant should be represented in the future to provide an accurate conclusion about GWAS. In contrast to our study, *G2964A* polymorphism has not been highlighted in any of the GWA studies on asthma. Several limitations were observed in our current study. First, the meta-analysis only considered the published studies enrolled from the chosen databases; therefore, results from unpublished studies may have been missed and could result in a potential bias. To minimize publication bias, a comprehensive search strategy was used, such as setting no language restriction, enrolling as many databases as possible, and so on. However, data from a conference article were still unavailable [28]. Second, we could not perform enough subgroup analysis because of the moderate number of studies and the relatively small scale of sample sizes included in our study. Third, the studies enrolled in our analysis were largely from a Caucasian population or an Asian population. Although subgroup analysis of the Asian population showed significant similar results to the overall population, associations of *STAT6* polymorphisms with other uninvestigated populations such as African populations should be carefully explained or predicted. Fourth, differences in most of the *G2964A* polymorphisms changed from moderate levels to significant levels after the study of Ding *et al*. was excluded [9]. This study satisfied the inclusion criteria and was in Hardy-Weinberg equilibrium. Thus, the results should be carefully interpreted after the study of Ding *et al*. was excluded.

In conclusion, our present study reported for the first time a comprehensive meta-analysis to determine the association between the *STAT6* gene polymorphisms and the risk of asthma based on data published until December 8, 2012. The *S* allele (*GT_13_* and *GT_14_*) of the *GT* repeat polymorphism indicates significant risks of asthma, whereas the *G2964A* polymorphism is possibly not associated with the susceptibility to asthma.

## Supporting Information

Figure S1 **Association between** ***G2964A*** **polymorphism in** ***STAT6*** **with the risk of asthma after removal of study of Ding** ***et al*** **.** The results were shown by forest plots**.** Each study is shown by the first author name, year of publication, individual and overall ORs (odds ratio) and 95% CI (confidence intervals). Box and horizontal line represent OR and 95% CI of the corresponding study, and the diamond represents the overall OR and 95% CI. (A) *AA* vs. *AG*+*GG* excluding Ding’s study in Chinese population, fixed-effects model; (B) *A* vs. *G* excluding Ding’s study in Chinese populations, fixed-effects model; (C) *AA* vs. *GG* excluding Ding’s study in Chinese population, fixed-effects model; (F) *AA* vs. *AG* excluding Ding’s study in Chinese population, fixed-effects model.
(TIF)

Table S1 **Sensitivity analysis and publication bias test results of different genetic models in GT repeat polymorphism of STAT6 gene.**
(DOC)

Table S2 **Sensitivity analysis and publication bias test results of different genetic models in G2964A polymorphism of STAT6 gene.**
(DOC)
