# Supplementary figures and images for: The Correlation Analysis of Two Common Polymorphisms in STAT6 Gene and the Risk of Asthma: A Meta-Analysis
Source: PLoS One. 2013 Jul 4;8(7):e67657. doi: 10.1371/journal.pone.0067657 (PMC3701693; doi:10.1371/journal.pone.0067657)

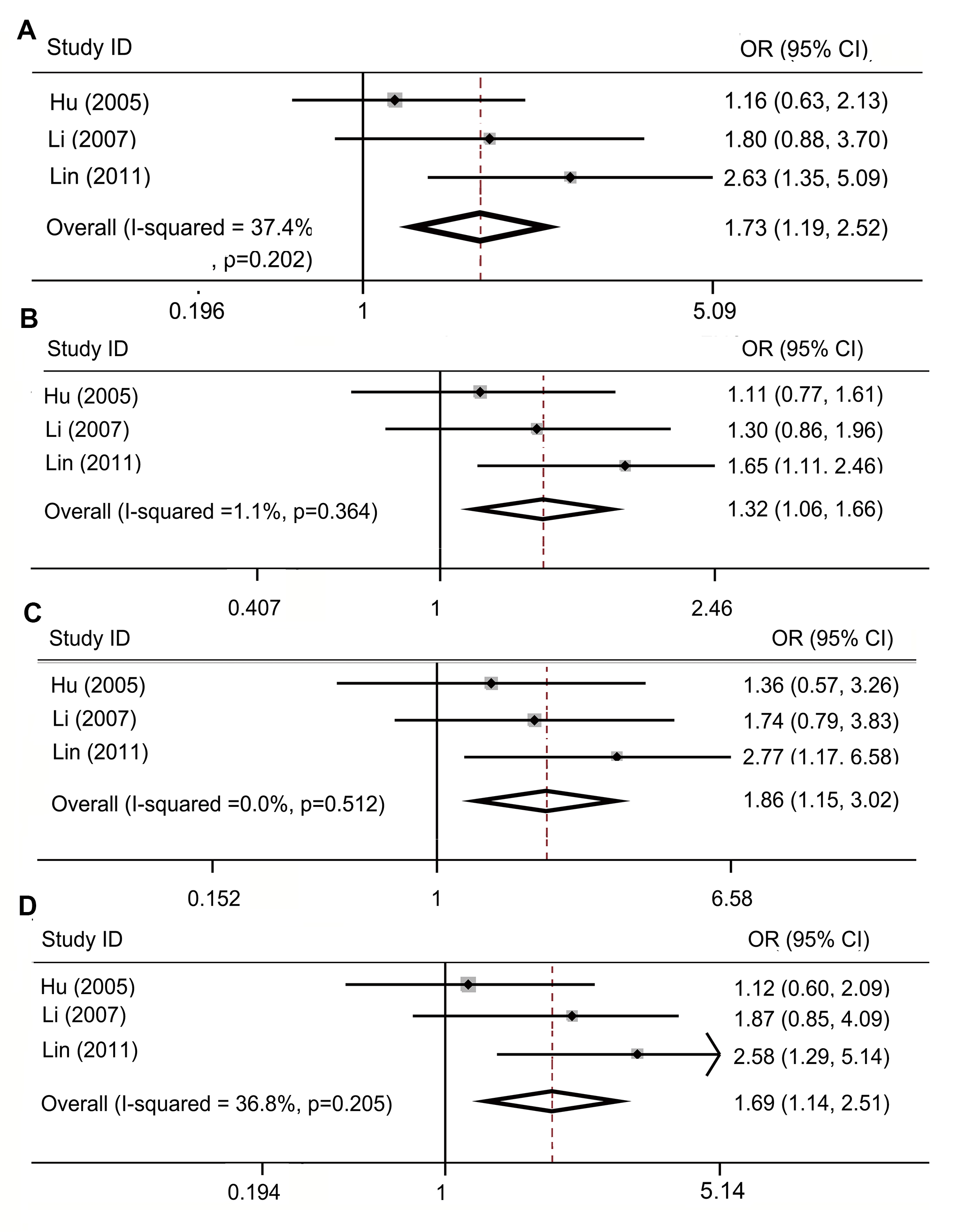

Supplement: Figure S1 — Association between G2964A polymorphism in STAT6 with the risk of asthma after removal of study of Ding et al . The results were shown by forest plots. Each study is shown by the first author name, year of publication, individual and overall ORs (odds ratio) and 95% CI (confidence intervals). Box and horizontal line represent OR and 95% CI of the corresponding study, and the diamond represents the overall OR and 95% CI. (A) AA vs. AG+GG excluding Ding’s study in Chinese population, fixed-effects model; (B) A vs. G excluding Ding’s study in Chinese populations, fixed-effects model; (C) AA vs. GG excluding Ding’s study in Chinese population, fixed-effects model; (F) AA vs. AG excluding Ding’s study in Chinese population, fixed-effects model. (TIF) [file pone.0067657.s001.tif]
