# Supplementary material for: The Correlation Analysis of Two Common Polymorphisms in STAT6 Gene and the Risk of Asthma: A Meta-Analysis
Source: PLoS One. 2013 Jul 4;8(7):e67657. doi: 10.1371/journal.pone.0067657 (PMC3701693; doi:10.1371/journal.pone.0067657)
Supplement: Table S1 — Sensitivity analysis and publication bias test results of different genetic models in GT repeat polymorphism of STAT6 gene. (DOC) [file pone.0067657.s002.doc]

**Table S1. Sensitivity analysis and publication bias test results of different genetic models in *GT* repeat polymorphism of *STAT6* gene.**

| **Genetic model /Study omitted**  **/Reference** | **OR** | **95% CI** | | **Publication bias (p)** | |
| --- | --- | --- | --- | --- | --- |
|  |  | **Lower limit** | **Upper limit** | **Begg’s test** | **Egger’s test** |
| *S* vs. *L* |  |  |  | 0.462 | 0.750 |
| Gao (2004) | 1.4477426 | 1.2038802 | 1.7410027 |  |  |
| Shao (2004) | 1.389941 | 1.1526451 | 1.6760893 |  |  |
| Suzuki (2004) | 1.4908055 | 1.2252374 | 1.8139352 |  |  |
| Hu (2005) | 1.3799264 | 1.1478297 | 1.6589542 |  |  |
| Wang (2011) | 1.5362453 | 1.27673 | 1.8485112 |  |  |
| *GT_13_* vs. *GT_15_* |  |  |  | 0.806 | 0.853 |
| Gao (2004) | 1.3793471 | 1.1380249 | 1.6718425 |  |  |
| Shao (2004) | 1.2998882 | 1.0676659 | 1.58262 |  |  |
| Suzuki (2004) | 1.4128041 | 1.1482427 | 1.7383218 |  |  |
| Hu (2005) | 1.362555 | 1.1250109 | 1.6502562 |  |  |
| Wang (2011) | 1.4687636 | 1.2081943 | 1.7855294 |  |  |
| *GT_13_* vs. *GT_14_* |  |  |  | 0.806 | 0.474 |
| Gao (2004) | 0.4100564 | 0.20240348 | 0.83074785 |  |  |
| Shao (2004) | 0.6579563 | 0.36694782 | 1.1797496 |  |  |
| Suzuki (2004) | 0.6367567 | 0.35509989 | 1.141817 |  |  |
| Hu (2005) | 0.7777809 | 0.3937529 | 1.5363525 |  |  |
| Wang (2011) | 0.5955784 | 0.31874002 | 1.1128622 |  |  |
| *GT_15_* vs. *GT_16_* |  |  |  | 0.308 | 0.405 |
| Gao (2004) | 0.8877889 | 0.61355512 | 1.284594 |  |  |
| Shao (2004) | 1.1956807 | 0.80332738 | 1.7796635 |  |  |
| Suzuki (2004) | 1.0955066 | 0.54788699 | 2.1904786 |  |  |
| Hu (2005) | 0.9938731 | 0.5463602 | 1.8079352 |  |  |
| Wang (2011) | 1.024946 | 0.66311826 | 1.5842036 |  |  |

OR: odds ratio; 95% CI: 95% confidence interval; *S*: short allele, allele ≤ 14 *GT* repeats; *L*: long allele, allele ≥ 15 *GT* repeats. *GT_13_*, *GT_14_*, *GT_15_*, *GT_16_* were abbreviations of the alleles with repeat number 13, 14, 15 and 16 respectively.

**References**

1. Gao PS, Heller NM, Walker W, Chen CH, Moller M, et al. (2004) Variation in dinucleotide (GT) repeat sequence in the first exon of the STAT6 gene is associated with atopic asthma and differentially regulates the promoter activity in vitro. Journal of Medical Genetics 41: 535-539.

2. Hasegawa K, Yang X, Endo H, Takayanagi R, Nakazawa C, et al. (2004) Linkage and association of childhood asthma with the chromosome 12 genes. Journal of Human Genetics 49: 115-122.

3. Suzuki M, Arakawa H, Kobayashi Y, Tamura K, Mochizuki H, et al. (2004) Signal transducers and activators of transcription 6 (Stat6) variants in childhood and adult asthma. Allergology International 53: 241-244.

4. Hu JH, Wu JM, Cui TP, Li YR, Lou DD (2005) Study on the correlation between STAT6 gene polymorphism and atopic asthma in Chinese Han population of Hubei province. Chinese Journal of Microbiology and Immunology 25: 243-247.

5. Wang Q, Bai XT, Xu DQ, Li H, Xu CY, et al. (2011) Association of polymorphisms of STAT6 and SO2 with Chinese Childhood Asthma: A case-control study. Biomedical and Environmental Sciences 24: 670-677.
