# Supplementary material for: The Correlation Analysis of Two Common Polymorphisms in STAT6 Gene and the Risk of Asthma: A Meta-Analysis
Source: PLoS One. 2013 Jul 4;8(7):e67657. doi: 10.1371/journal.pone.0067657 (PMC3701693; doi:10.1371/journal.pone.0067657)
Supplement: Table S2 — Sensitivity analysis and publication bias test results of different genetic models in G2964A polymorphism of STAT6 gene. (DOC) [file pone.0067657.s003.doc]

**Table S2. Sensitivity analysis and publication bias test results of different genetic models in *G2964A* polymorphism of *STAT6* gene.**

| **Genetic model /Study omitted**  **/Reference** | **OR** | **95% CI** | | **Publication bias (p)** | |
| --- | --- | --- | --- | --- | --- |
|  |  | **Lower limit** | **Upper limit** | **Begg’s test** | **Egger’s test** |
| *GG* vs. *AA*+*AG* |  |  |  | 1.00 | 0.959 |
| Gao (2000) | 1.0817782 | .82040251 | 1.4264267 |  |  |
| Gao (2000) | 1.0155002 | .79088794 | 1.3039025 |  |  |
| Tamura (2003) | 1.0413429 | .81146176 | 1.3363478 |  |  |
| Hu (2005) | 1.1296005 | .88215709 | 1.4464512 |  |  |
| Li (2007) | 1.1387922 | .88132786 | 1.4714702 |  |  |
| Ding (2010) | 1.0845513 | .84589179 | 1.3905462 |  |  |
| Lin (2011) | 1.1558792 | .90137775 | 1.4822384 |  |  |
| *GG* vs. *AA*+*AG* ^#^ |  |  |  | 0.734 | 0.856 |
| Hu (2005) | .88215465 | .60287444 | 1.2908108 |  |  |
| Li (2007) | .85876117 | .56635698 | 1.3021306 |  |  |
| Ding (2010) | .7925857 | .5380345 | 1.1675684 |  |  |
| Lin (2011) | .92666792 | .63033405 | 1.3623148 |  |  |
| *AG*+*GG* vs. *AA* ^#^ |  |  |  | 0.734 | 0.543 |
| Hu (2005) | .65324294 | .31490386 | 1.3551004 |  |  |
| Li (2007) | .75537602 | .3845733 | 1.483704 |  |  |
| Ding* (2010) | .57627458 | .35564944 | .93376329 |  |  |
| Lin (2011) | .87515066 | .55868415 | 1.3708795 |  |  |
| *G* vs. *A*^#^ |  |  |  | 0.734 | 0.303 |
| Hu (2005) | .82443725 | .65752201 | 1.0337248 |  |  |
| Li (2007) | .86623522 | .6965649 | 1.0772341 |  |  |
| Ding* (2010) | .75634993 | .60392941 | .94723854 |  |  |
| Lin (2011) | .93324381 | .74887697 | 1.1630001 |  |  |
| *AG* vs. *AA*^#^ |  |  |  | 0.308 | 0.270 |
| Hu (2005) | .64912216 | .31452232 | 1.339681 |  |  |
| Li (2007) | .76695674 | .39786216 | 1.4784584 |  |  |
| Ding* (2010) | .58404936 | .35250848 | .96767503 |  |  |
| Lin (2011) | .88372926 | .5640811 | 1.3845126 |  |  |
| *GG* vs. *AA*^#^ |  |  |  | 1.000 | 0.719 |
| Hu (2005) | .66556081 | .41670157 | 1.0630418 |  |  |
| Li (2007) | .72491428 | .44668859 | 1.1764364 |  |  |
| Ding* (2010) | .53690977 | .33099812 | .87091765 |  |  |
| Lin (2011) | .8220624 | .51225344 | 1.3192427 |  |  |
| *GG* vs. *AG*^#^ |  |  |  | 0.089 | 0.268 |
| Hu (2005) | 1.0293184 | .68626717 | 1.543854 |  |  |
| Li (2007) | .9399926 | .60994987 | 1.4486208 |  |  |
| Ding (2010) | .95308451 | .63220428 | 1.43683 |  |  |
| Lin (2011) | .99586687 | .66238891 | 1.4972334 |  |  |

**^#^**the subgroup of Chinese population; *the remove of this study generated a significant difference; OR: odds ratio; 95% CI: 95% confidence interval.

**References**

1. Gao PS, Mao XQ, Roberts MH, Arinobu Y, Akaiwa M, et al. (2000) Variants of STAT6 (signal transducer and activator of transcription 6) in atopic asthma. Journal of Medical Genetics 37: 380-382.

2. Tamura K, Suzuki M, Arakawa H, Tokuyama K, Morikawa A (2003) Linkage and Association Studies of STAT6 Gene Polymorphisms and Allergic Diseases. International Archives of Allergy and Immunology 131: 33-38.

3. Hu JH, Wu JM, Cui TP, Li YR (2005) Correlation of the gene polymorphism at position 2964(G/ A) in 3'-untranslated region of STAT 6 gene with asthma and serum IgE in Chinese Han population of Hubei. Chinese Journal of Clinical Laboratory Science 23: 9-12.

4. Li Y, Wu B, Xiong H, Zhu C, Zhang L (2007) Polymorphisms of STAT-6, STAT-4 and IFN-γ genes and the risk of asthma in Chinese population. Respiratory Medicine 101: 1977-1981.

5. Ding YP, He HW, Yao HX, Lin L, Shi HF (2010) Relationship between STAT6 gene polymorphism and bronchial asthma in Li-nationality people in Hainan. Chinese General Practice 13: 1765-1767.

6. Lin RJ, Liu DX, Sui AH (2012) Relationship between signal transducers and activators of transcription 6 gene polymorphism and genetic susceptibility of bronchial asthma in children. Journal of Applied Clinical Pediatrics 27: 1258-1260.
